# Cardiac Amyloidosis: State-of-the-Art Review in Molecular Pathology

**DOI:** 10.3390/cimb46100684

**Published:** 2024-10-16

**Authors:** Cecilia Salzillo, Renato Franco, Andrea Ronchi, Andrea Quaranta, Andrea Marzullo

**Affiliations:** 1 Department of Experimental Medicine, PhD Course in Public Health, University of Campania “Luigi Vanvitelli”, 80138 Naples, Italy; 2 Department of Precision and Regenerative Medicine and Ionian Area, Pathology Unit, University of Bari “Aldo Moro”, 70121 Bari, Italy; a.quaranta057@gmail.com; 3 Department of Mental and Physical Health and Preventive Medicine, Pathology Unit, University of Campania “Luigi Vanvitelli”, 80138 Naples, Italy; renato.franco@unicampania.it (R.F.); andrea.ronchi@unicampania.it (A.R.)

**Keywords:** cardiac amyloidosis, cardiomyopathies, heart diseases, cardiovascular diseases, molecular pathology, molecular mechanisms

## Abstract

Amyloidosis refers to a group of diseases caused by extracellular deposits of misfolded proteins, which alter tissue function and structure, potentially affecting any organ. The term “amyloid” was introduced in the 19th century and later associated with pathological protein deposits. Amyloid fibrils, which are insoluble and resistant to degradation, originate from soluble proteins that undergo misfolding. This process can be triggered by several factors, such as aging, elevated protein concentrations, or pathogenic variants. Amyloid deposits damage organs both by disrupting tissue architecture and through direct cytotoxic effects, leading to conditions such as heart failure. Amyloidosis can be classified into acquired or inherited forms and can be systemic or localized. Diagnosing cardiac amyloidosis is complex and often requires tissue biopsies, which are supported by Congo Red dye staining. In some cases, bisphosphonate bone scans may provide a less invasive diagnostic option. In this state-of-the-art review, we focus on the most common forms of cardiac amyloidosis, from epidemiology to therapy, emphasizing the differences in molecular mechanisms and the importance of pathological diagnosis for appropriate treatment using a multidisciplinary approach.

## 1. Introduction

Amyloidosis is a heterogeneous group of diseases caused by the deposition of toxic insoluble fibrillar beta-sheet protein aggregates in different tissues, resulting in functional and morphological changes [1], representing a major diagnostic and management challenge requiring a multidisciplinary team.

The term “amyloid” derives from the Latin “amylum”, which literally translates to “starch”, and was introduced for the first time by the German botanist Matthias Schleiden in his research to indicate the presence of starch in plants [2].

The first use of the term “amyloid” in relation to human pathophysiology was attributed to the German doctor Rudolf Virchow, who, in the 19th century, used it to describe a tissue anomaly that showed a positive reaction to iodine in the cerebral gray matter of subjects suffering from dementia, who believed that these structures had the same composition as cellulose and called them “corpora amylacea” [2].

In the second half of the 19th century, it became clear that they consisted of proteins. In 1922, it was discovered that amyloid deposits show birefringence under a polarized light microscope and increase in intensity after staining with Congo red dye; thus, congophilia with birefringence Apple green became the first criterion for diagnosing amyloidosis [2].

Beginning in the 1950s, advances in electron microscopy made it possible to characterize amyloid fibrils from an ultrastructural point of view, and the fibrillar ultrastructure was incorporated into the histopathological definition of amyloid. Over 20 biochemically distinct forms of amyloid have been identified, each associated with a unique clinical syndrome [2].

Amyloids can accumulate in various organs, such as the heart, blood vessels, liver, spleen, kidneys, and nerves, where they cause cardiomyopathy, hepatomegaly, proteinuria, macroglossia, autonomic dysfunction, ecchymosis, neuropathy, renal failure, hypertension, and corneal and vitreous abnormalities [1].

Specifically, the extracellular deposition of amyloid fibrils in the myocardium leads to cardiac amyloidosis (CA) [3], which is the most frequent type of restrictive cardiomyopathy [4,5] and is a progressive, often fatal disease, causing heart failure, severe aortic stenosis, and unexplained left ventricular hypertrophy [6,7,8].

CA is rare and represents the main cause of mortality in patients with systemic amyloidosis [9]. The prevalence of CA varies according to the etiological type, with an increase from 8–17 per 100,000 person-years in recent years [10].

The International Society of Amyloidosis (ISA) has identified over 40 proteins capable of forming amyloids, but only 10 cause significant deposition in the heart, leading to CA [11]. Among these, light chain (AL) and transthyretin (ATTR) amyloidosis are the most common forms in clinical practice, and distinguishing the type of amyloid is crucial since the differences in prognosis and treatment between the various types of CA are relevant [5,12].

Furthermore, correct amyloid typing is essential to identify hereditary forms of CA, thus allowing targeted genetic counseling to be offered and other family members who may be affected or at risk to be promptly identified.

## 2. Materials and Methods

The state-of-the-art review was conducted in PubMed and Scopus with the following search keywords: “*Cardiac amyloidosis*” OR “*Light-chain amyloidosis*” OR “*Transthyretin amyloidosis*” OR “*Amyloidogenesis*” OR “*Molecular mechanisms AND cardiac amyloidosis*”, with inclusion criteria for primary studies, secondary studies, and English language. Additionally, Google Scholar was used for gray literature.

## 3. Cardiac Amyloidosis

To date, the ISA recognizes 40 different amyloidogenic proteins in humans, 10 of which are deposited in the heart, causing cardiac amyloidosis and thus cardiomyopathy [11].

Clinically, types of amyloidosis are classified according to whether they are acquired or hereditary and whether they are systemic or localized to a specific organ.

Acquired amyloidosis includes some of the most frequently encountered forms of systemic amyloidosis, such as light-chain amyloidosis (AL), wild-type transthyretin amyloidosis (ATTRwt), serum amyloid A (AA) amyloidosis, and leukocyte-derived chemotaxis 2 (ALECT2).

Hereditary amyloidosis includes systemic pathologies frequently associated with cardiomyopathies, neuropathies, and nephropathies, with variant transthyretin amyloidosis (ATTRv) being the most frequent, and other forms of hereditary systemic amyloidosis occur owing to pathogenic variants in the fibrinogen alpha chain, apolipoprotein A I, lysozyme, and jasmine.

The most frequent amyloidosis is AL, followed by ATTR, and Table 1 summarizes the red flags [12,13] to suspect cardiac amyloidosis (CA). Briefly, the evaluation of CA is recommended according to the European Society of Cardiology (ESC) when there is a left ventricular wall thickness ≥ 12 mm and at least one red flag [12] instead of the American Heart Association (AHA) when red flags are present regardless of thickness [13].

A study by Muller et al. examined 114 patients with ATTR amyloidosis to verify the correlation between cardiac wall thickness measured by echocardiography and amyloid accumulation assessed by bone scintigraphy. The results showed that a significant portion of patients, approximately 12%, had no wall thickening (IWT) at the time of diagnosis, contradicting the current criterion [14].

Furthermore, a strong correlation between wall thickness and amyloid accumulation was not found, suggesting that bone scanning can detect amyloidosis even before the onset of IWT. This implies that wall thickness > 12 mm may not be a reliable marker for all patients, and that further studies are needed to improve diagnostic models [14].

### 3.1. Light-Chain Amyloidosis

Light chain amyloidosis (AL), previously known as primary amyloidosis, is the most frequent form of systemic amyloidosis, accounting for approximately 70% of all patients affected by these diseases [15]. AL is caused by the clonal production of kappa (κ) or lambda (λ) light chains or their fragments by plasma cells, which aggregate and are deposited in various tissues and organs [15].

It is a relatively rare condition, with an estimated global incidence of 5.1–12.8 cases per million person-years [16] and a minimum incidence of 8–12 per million [17], and it is the cause of death in 0.58 of the 1000 deaths registered [18]. The median age at diagnosis is 76 years [17], and there appear to be geographic and racial disparities in diagnosis [19].

In AL amyloidosis, clonal plasma cells in the bone marrow produce amyloidogenic immunoglobulins, and nearly any plasma cell dyscrasia can be complicated by AL amyloidosis [20]. In 80% of cases, the offending chain produced by the B-cell clone is lambda, whereas in 20% of cases, it is kappa [21]. Pathogenic variants can occur in the gene encoding the variable region of the light chain, leading to a reduction in the stability of the protein, which results in a higher concentration of circulating free light chains [22]. Even in the prefibrillar state, these complexes can induce proteotoxicity and increase cellular oxidative stress, resulting in the generation of oxygen free radicals and the alteration of intracellular calcium fluxes [23].

The organs most affected are the heart in 70–80% of patients and the kidneys in 60–70% of patients [24].

Cardiac involvement is usually dominated by diastolic heart failure with preserved ejection fraction [25], which manifests as syncope, dizziness, orthostatic hypotension, and asthenia and is the main cause of death [24].

Early signs of cardiac AL include low ECG voltage, ventricular thickening, diastolic dysfunction, and poor atrial contractility, increasing the risk of thrombi and thromboembolic complications [24], and bradyarrhythmias often precede terminal heart failure [26].

The second most affected organ is the kidney, with proteinuria being the most prevalent clinical manifestation [27], followed by hypoalbuminemia, secondary hyperlipidaemia, and oedema. Occasionally, renal failure occurs in the absence of proteinuria as a result of interstitial or vascular amyloid deposition [28].

The peripheral nervous system may also be affected by carpal tunnel syndrome and nondiabetic sensory polyneuropathy [25]. In the advanced stages, pathognomonic signs such as periorbital purpura and macroglossia can appear, but with low sensitivity; nevertheless, it is highly specific for the presence of disease [25]. Periorbital purpura is caused by the accumulation of amyloid in the blood vessels, making them fragile and susceptible to microtrauma, such as rubbing the eyes. Macroglossia is caused by the deposition of amyloid in the tongue tissue, which can interfere with swallowing, phonation, and even breathing, making it a clinically significant symptom of the disease.

Free light chain (FLC) testing can detect elevated levels and altered ratios of light chains, which are present in 98% of patients with systemic AL, although a monoclonal immunoglobulin cannot be detected by conventional methods. Importantly, however, this test is not specific for AL, as monoclonal FLCs can also be found in patients with other types of B-cell malignancies.

Staging for AL is determined by assessing the presence and severity of cardiac damage, which is accomplished by measuring cardiac troponin T (TnT) and beta-terminal pro natriuretic peptide (NT–proBNP) levels in the blood [29,30].

Survival of patients with AL systemic amyloidosis is closely linked to the severity of cardiac dysfunction at diagnosis. A late diagnosis, with advanced heart damage, reduces the median survival to 3–6 months, whereas those without cardiac involvement can live for many years [24].

### 3.2. Transthyretin Amyloidosis

Transthyretin amyloidosis (ATTR) occurs when the transthyretin (TTR) protein, which is typically synthesized by the liver, undergoes pathological misfolding and aggregates into amyloid fibrils and is deposited in multiple tissues, causing irreversible damage [31].

TTR is a protein that transports the thyroid hormone thyroxine (T4) and retinol-binding protein (vitamin A) and is produced mainly in the liver but also in the choroid plexus and retinal pigment epithelium [32].

ATTR includes two main forms: ATTRv or transthyretin-related hereditary amyloidosis and ATTRwt or senile systemic amyloidosis (SSA).

ATTRv is an autosomal dominant inherited disorder characterized by pathogenic variants in the *TTR* gene that present with misfolding of the protein. The mutation presents 2 predominant phenotypes, such as primarily neurological involvement (TTR-FAP, familial amyloid polyneuropathy) or primarily cardiac involvement (TTR-FAC, familial amyloid cardiomyopathy) [33].

ATTRwt is the most prevalent form of transthyretin amyloid cardiomyopathy, primarily affecting individuals over the age of 60 with an average age of 80 years and is characterized by the natural tendency of wild-type TTR to aggregate and form harmful amyloid fibrils [34].

Cardiac ATTR occurs in a significant proportion of patients with certain cardiac conditions; up to 16% of patients with aortic stenosis undergo transcatheter aortic valve replacement, and 19% of patients with severe mitral regurgitation undergo transcatheter mitral repair [35].

A 2013 study in the UK revealed that the annual incidence of systemic amyloidosis is approximately 8 new cases per million people per year, with 7% and 10% of cases attributed to ATTRv and ATTRwt, respectively [36].

In ATTR, protein aggregation is caused by reduced folding stability, physiological aging, and amyloidogenic pathogenic variants, which are typically missense and destabilize the original TTR, resulting in the breakdown of the tetramer into individual monomers that subsequently misfold and form amyloid fibrils [37]. Diffusible oligomers can bind to receptors on cell membranes, including the receptor for advanced glycation end products (RAGE). This binding can trigger a series of intracellular inflammatory responses leading to calcium influx, endoplasmic reticulum stress, and apoptosis. Amyloid fibrils can cause tissue damage via direct compression or obstruction, which can be observed in conditions such as carpal tunnel syndrome, vitreous opacities, and spinal canal stenosis [38].

Amyloid fibrils can invade cardiovascular structures in both forms of ATTR, resulting in thickening of the left and right ventricular walls and the interventricular septum [39], and symptoms of restrictive cardiomyopathy and/or heart failure with preserved ejection fraction may occur.

ATTRv amyloidosis is characterized by slowly progressive peripheral neuromotor and/or autonomic neuropathy, which may involve the heart, central nervous system, eyes, and kidneys [40].

Most people develop heart problems after age 50, such as arrhythmias (atrioventricular block, sick sinus syndrome, and atrial fibrillation), due to amyloid deposition in the heart. This deposition leads to progressive restrictive cardiomyopathy and heart failure, with diastolic ventricular dysfunction preceding systolic dysfunction. In some cases, cardiomyopathy is the main symptom without the presence of peripheral neuropathy [40].

A typical electrocardiogram revealed a pseudoinfarction pattern with a prominent Q wave due to amyloid deposition in the left ventricular wall. An echocardiogram revealed left ventricular hypertrophy with preserved systolic function and thickened walls with a “granular, glistening appearance” [40].

The most prevalent genetic ATTR mutation is *p.Val30Met* [41], which is often associated with polyneuropathy, the most common phenotype of ATTRv, ranging from peripheral neuropathy to carpal tunnel syndrome and spinal stenosis. Additionally, musculoskeletal problems may include weakness, fatigue, spontaneous bicep tendon rupture (Popeye sign), and degenerative joint disorders [42].

The advancement of diphosphonate scintigraphy has facilitated the diagnosis of both ATTRwt and ATTRv, reducing the need for invasive biopsy diagnosis in selected cases [42,43]. Mass spectrometry is the gold standard for defining amyloid type, whereas immunohistochemistry or immunoelectron microscopy are commonly used for amyloid typing. The diagnosis is confirmed if amyloid deposits within an extracardiac biopsy are accompanied by echocardiographic features characteristic of cardiac amyloidosis in the absence of any alternative cause for increased left ventricular wall thickness [12].

CA-ATTR has a better prognosis than CA-AL does, although ATTR is a progressive condition with limited treatment options. However, early diagnosis is crucial for prompt treatment of cardiac and neurological symptoms, as treatment is more effective in the early stages of the disease [44].

## 4. Diagnosis in Pathology

Cardiac amyloidosis currently represents a diagnostic challenge, both owing to its subtle clinical presentation and heterogeneous etiopathogenesis, resulting in various prognostic and therapeutic implications.

The most reliable method for diagnosing amyloidosis is tissue biopsy, with the exception of ATTR, which can be diagnosed without tissue biopsy, provided that a number of criteria are met [43]. This procedure allows the identification and classification of amyloid deposits via histochemical and immunohistochemical staining.

In the case of cardiac amyloidosis, the diagnosis is based on periumbilical fat biopsy via abdominal subcutaneous fat aspiration and endomyocardial biopsy.

Periumbilical fat biopsy is commonly used because of its minimally invasive nature, but the main limitation of this procedure is its relatively low diagnostic sensitivity [45], especially in the ATTRwt, and therefore it cannot be used to exclude it [46].

Endomyocardial biopsy has increased sensitivity, but it is an invasive procedure that can be performed only in specialized medical centers.

**Figure 1:**
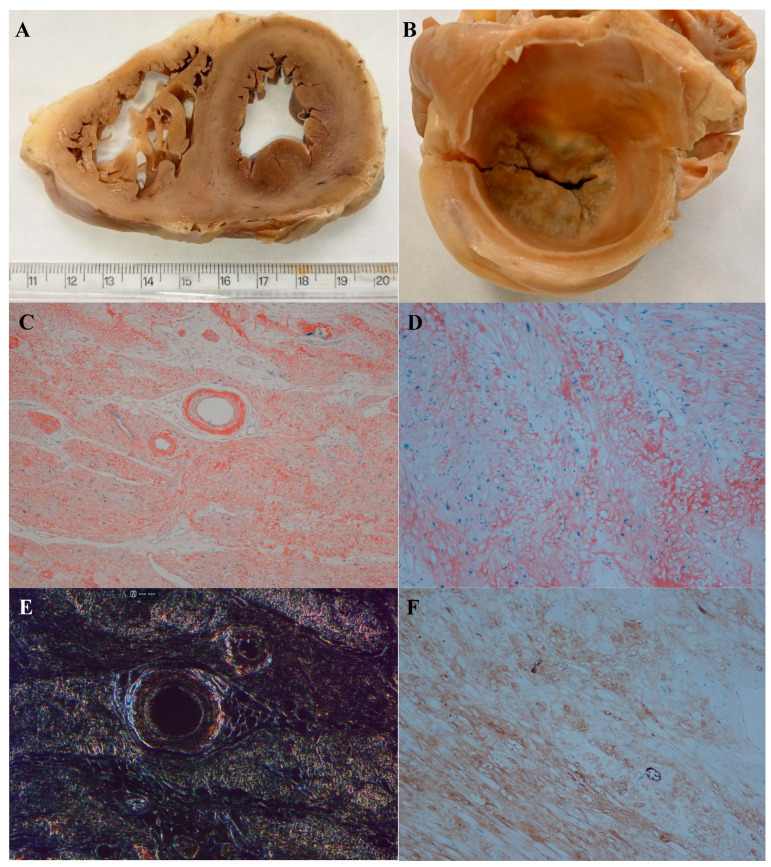
Cardiac amyloidosis AL. (**A**) Macroscopic transverse section of right and left ventricular myocardium with pale and waxy appearance. (**B**) Macroscopic transverse section of the left atrial myocardium with a pale and waxy appearance. (**C**) Congo Red staining demonstrates a diffuse perivascular, interstitial, and pericellular amyloid deposition (magnification 4×). (**D**) At higher magnification (10×), the pericellular and interstitial deposition of congophilic substance (amyloid) becomes more evident, sometimes forming small nodular aggregates. (**E**) Congo Red staining under polarized light shows a green apple birefringence demonstrating interstitial and perivascular amyloid deposition (magnification 10×). (**F**) Strong immunohistochemical evidence of kappa light chains in the myocardial interstitium with a pericellular and diffuse pattern (magnification 10×).

Amyloid deposits accumulate primarily in the myocardium, which appears macroscopically pale and waxy, with a thickened and rubbery consistency, and may also affect the atria, pericardium, endocardium, and vascular microsystem, with a grainy or sandpaper-like appearance (Figure 1A,B). Amyloid deposits can be observed as amorphous, eosinophilic deposits on histological sections stained with hematoxylin–eosin (HE), and importantly, HE staining is not specific to amyloid deposits, as hyaline changes or sclerosis may result in a similar appearance.

To confirm amyloid deposition, Congo Red staining is commonly used (Figure 1C,D) [45]. Congo Red-stained amyloids have an orange-red color when viewed under a light microscope and an apple-green birefringence when viewed under polarized light during histological studies (Figure 1E) [45].

However, it is important to note that Congo Red has several limitations. Some tissue components, such as hyaline tissues, mucous, fibrinoids, or elastics, can “absorb” Congo Red, compromising its specificity. Positively stained tissue elements, such as elastin and collagen, can generally be identified on the basis of their appearance or location [6].

If Congo Red is negative but a strong clinical or morphological suspicion of amyloidosis persists, it is advisable to repeat staining in other tissue sections [6].

If Congo Red is positive but there is no histological evidence of extracellular deposits, it is advisable to compare the morphological results with tissue sites showing birefringence or to use other stains such as thioflavin T, thioflavin S, or Alcian blue; another option is the Trichrome by Azan Mallory. Amyloid with thioflavin appears yellow, turning red with increasing fluorescence, while with Azan Mallory Trichrome it is bluish gray [6].

Amyloid deposits in the heart are found in both the myocardium and cardiac vessels with variable distribution [6].

Initially, CA deposits may be focal and difficult to detect. As they become more extensive, they may follow different infiltrative patterns. In particular, in the myocardium, amyloid can surround cardiomyocytes individually, causing atrophy or forming nodular aggregates that alter the structure of the tissue. AL amyloid deposits tend to be pericellular and accompanied by inflammation, while ATTR deposits are more nodular and irregular. In blood vessels, amyloid can affect arteries, veins, and capillaries, causing blockages.

Furthermore, deposition may be associated with secondary histological changes, such as inflammation and ischemic damage [6].

Once amyloid deposits have been confirmed, fibril subtyping is essential [45].

Immunohistochemistry (IHC) is widely used with antibodies against TTR, AA, lambda, and kappa (Figure 1F). The limitations of IIC are the limited availability of antibodies for all amyloid subtypes and the potential lack of sensitivity and specificity, especially kappa or lambda light chains, making typing of AL amyloid difficult [45].

Immunofluorescence (IF) uses antibodies labeled with fluorescent dyes and observes the results with a fluorescence microscope on frozen sections to avoid problems of autofluorescence and alterations due to fixation. IF has fewer problems with antibody reactivity and background staining but is limited by variable results like IIC [45].

Immunoelectron microscopy (IEM) combines IHC with ME to identify proteins in amyloid fibrils using gold-labeled antibodies with high sensitivity and specificity. Its use is limited by the availability and lack of specialized expertise [45].

When IIC and IEM are inconclusive, laser capture microdissection followed by tandem mass spectrometry (LCM–MS) is a highly effective technique to characterize proteins in amyloid fibrils. This technique analyzes amyloid deposits on Congo red-stained paraffin sections, identifying the protein type by mass spectrometry. It also has a sensitivity and specificity that significantly exceed IHC [45].

The process of obtaining a myocardial biopsy can be invasive. Therefore, in cases where there are typical echocardiographic findings, together with grade 2 or 3 myocardial radiotracer uptake on diphosphonate bone scans, ATTR cardiac amyloidosis can be diagnosed without the need for histological examination. It is also crucial to perform a series of tests to exclude clonal dyscrasia, which include a serum-free light chain assay and serum and urine protein electrophoresis with immunofixation [12].

While non-invasive methods can diagnose ATTR cardiac amyloidosis, histological confirmation and precise typing of amyloid deposits remain essential for many patients with ATTR, as well as for all patients with AL amyloidosis and other rare forms of cardiac amyloidosis.

Table 2 describes the steps for the diagnosis of cardiac amyloidosis.

## 5. Molecular Pathology

### 5.1. Structure

The abnormal accumulation of insoluble polymeric fibrillar proteins outside of cells, in tissues, blood vessels, and organs is the hallmark of amyloidosis [47].

Amyloid fibrils are insoluble fibers that form due to incorrect folding during the assembly process of normally soluble proteins, making them resistant to degradation [48].

Unbranched fibril assemblies are structurally stable and composed predominantly of repeats of β-strands that are perpendicular to the fiber axis, forming a characteristic cross-β-sheet of indefinite length, specifically antiparallel β-strands that lie perpendicular to the long axis of the fibril [49,50].

Amyloids arise from the ordered assembly of numerous copies of a peptide or protein. These structures are observable via electron microscopy techniques and appear as elongated, unbranched filaments with diameters between 6 and 12 nm [51]. X-ray diffraction patterns of the fibers, characterized by the repetitive arrangement of crossed β-sheets, exhibit a meridional reflection of approximately 4.7 Å, indicating the distance between the β-strands, and an equatorial reflection between 6 and 11 Å, reflecting the distance between the stacked β-sheets [51]. This peculiar structure may confer several functions, which can be both beneficial and detrimental to the cell.

### 5.2. Amyloidogenesis

All forms of amyloidosis are characterized by the presence of deposits of insoluble protein fibrils originating from poorly folded proteins, and polypeptides can adopt alternative misfolded configurations, increasing their susceptibility to aggregation [52].

Several mechanisms can lead to the misfolding of proteins. In some cases, the protein itself may be intrinsically predisposed to develop a pathological conformation with age, as occurs in patients suffering from senile systemic amyloidosis due to wild-type transthyretin [53]. Furthermore, misfolding can occur when the concentration of protein precursors in the serum is high, as observed in long-term hemodialysis patients with high levels of beta-2-microglobulin [53].

Some proteins, defined as intrinsically misfolded, have at least one section that does not possess a stable secondary or tertiary structure, allowing them to change their conformation to better interact with ligands [54]. Among these proteins, apolipoprotein A I, A II and serum amyloid A (SAA) are frequently associated with the formation of amyloid deposits [54].

In the case of hereditary amyloidosis, the substitution of individual amino acids can compromise the biological function of misfolded proteins, favoring the formation of aggregates [53]. In Alzheimer’s disease, proteolytic modification of amyloid-beta precursor proteins contributes to pathology. In patients with AA amyloidosis, serum amyloid A protein, an acute-phase protein, may accumulate in several tissues [53]. These mechanisms can act alone or in combination with each other.

### 5.3. Molecular Mechanisms

Organ damage in the CA is not explained only by the displacement of normal parenchymal tissue due to amyloid deposits [55]. Indeed, atrophy and degeneration of cells around amyloid fibrils were observed in nerve and cardiac biopsy samples, suggesting that oligomer toxicity, inflammatory responses, oxidative stress, and extracellular matrix remodeling also contribute to tissue damage [56].

Table 3 summarizes the pathological mechanisms related to CA-AL and CA-ATTR, highlighting the different molecular pathways and cardiac alterations involved.

#### 5.3.1. Light Chain Amyloidosis

Amyloid fibrils in AL amyloidosis originate from monoclonal light chains of immunoglobulins produced by plasma cells. These light chains (LCs) weigh approximately 22–23 kDa and are composed of two regions with β-sheet structures, the variable domain (VL) and the constant domain (CL) [55]. VL is characterized by great variability due to gene recombination and somatic pathogenic variants, whereas CL presents minimal variations between the two light chain isotypes, κ and λ [55]. Both isotypes, κ and λ, form homodimers via disulfide bonds, and approximately 75% of monoclonal amyloidogenic LCs are of the λ isotype [57].

Studies in the literature [55,58,59] have demonstrated that the breakdown of the LC structure in the amyloid fibrils of patients with AL amyloidosis is a multistep mechanism.

In patients with severe cardiac involvement, approximately 50–70% [60], the presence of fibrils compromises ventricular relaxation by altering contractile function [55]. Soluble cardiotropic LCs are internalized into cardiomyocytes and cardiac fibroblasts and interact with intracellular proteins, causing oxidative stress, mitochondrial ultrastructural changes, and the activation of apoptosis [61] through p38 MAPK activation and a reduction in NAD(P)H-dependent oxidoreductase [62,63].

Furthermore, increased expression of MMP9 and TIMP-1 and significantly increased serum levels of MMP9 and TIMP-1 were observed in myocardial biopsy samples compared with those of patients with ATTR amyloidosis with the same level of left ventricular hypertrophy [64], suggesting significant activation of extracellular matrix proteolysis in AL cardiomyopathy [55].

#### 5.3.2. Transthyretin Amyloidosis

ATTRv is a systemic amyloidosis with autosomal dominant transmission characterized by a genetic mutation of TTR, of which more than 150 variants have been identified, the majority of which are amino acid pathogenic variants due to a nucleotide substitution [55,65].

Pathogenic variants in TTR cause instability of the TTR tetramer, leading to its dissociation into monomers, a crucial step in amyloid formation. Different amyloidogenic TTR variants, such as *D18G*, *V30M*, *L55P*, *H88R*, *Y114H*, *Y116S*, and *V122I*, have lower structural stability than w-t TTR and are responsible for different clinical phenotypes [55,60].

A highly unstable TTR is more likely to cause ocular, central, or peripheral nervous system symptoms, whereas a relatively stable TTR is more likely to cause cardiac symptoms [66].

The clinical phenotypes are primary polyneuropathy in *V30M*, cardiomyopathy in *V20I*, *V122I*, *L111M*, and *I68L*, and mixed in *E89Q* and *T60A*; of these, the most frequent mutation worldwide is *V122I*, which is responsible for familial cardiomyopathy, especially in individuals of African origin [55].

The *S52P* variant produces a fragment of TTR (residues 49–127) that rapidly aggregates into amyloid fibrils under the influence of shear stress from physiological fluids. This proteolysis-fibrillation process appears to be particularly relevant in the heart, where shear stress is highest, and the fragment is abundant in cardiac amyloids [67].

TTR *V30M* is vulnerable to plasmin-mediated proteolysis, and SerpinA1, a serine protease inhibitor, has been found to inhibit this process in vitro, increasing plasma TTR levels and cardiac amyloid deposition [68].

In contrast, some stabilizing TTR pathogenic variants, such as *R104H*, *A108V*, and *T119M*, have been reported [69], and it seems that *T119M* variant carriers might be protected against cerebrovascular diseases and have a longer life [70].

These variants can have different effects on the heart. In particular, amyloidogenic variants of TTR, such as *V122I*, activate mechanisms of oxidative stress and apoptosis in cardiac cells, resulting in the cytotoxicity of cardiomyocytes [71]. Amyloid fibers in ATTRv initially form in the basement membrane of cardiomyocytes and vascular smooth muscle cells, with a parallel increase in membrane components and cardiac amyloid deposition [55].

ATTRwt amyloidosis is a disease that affects mainly the heart, tendons, ligaments, kidneys, thyroid, peripheral nerves, and lungs; is frequent in men over 60 years of age; and is caused by TTR, which becomes unstable with aging, dissociates, and forms amyloid fibrils [55].

The precise mechanism of dissociation of TTR tetramers with age is unclear but may be due to posttranslational biochemical changes or the reduced capacity of chaperone proteins in the liver. Furthermore, there is a sex difference in the incidence of ATTRwt with cardiac symptoms, with a higher prevalence in men, although the reason for this difference is not yet understood [72].

### 5.4. Relevance of Molecular Diagnosis

Early and accurate diagnosis of CA is essential to properly manage the disease, as therapeutic options vary significantly depending on the type of amyloidosis; to this end, molecular diagnosis is of fundamental importance.

In ATTR, the most frequent form of CA, it is crucial to distinguish between ATTRv and ATTRwt, and in fact, through molecular diagnosis with advanced sequencing techniques, it is possible to identify specific mutations in the TTR gene that cause the hereditary variant. This distinction has significant therapeutic implications, as some drugs, such as gene silencers, can be particularly effective in hereditary forms. Furthermore, early diagnosis in mutation carriers can allow preventive management, anticipating the onset of the disease.

The use of advanced sequencing techniques, such as whole exome sequencing (WES), gene panels, and whole genome sequencing (WGS), has improved the ability to diagnose and characterize the disease in a precise and personalized way.

WES is a technique that focuses on the analysis of the coding regions of the genome, the exons, where most pathogenic variants are found, and is particularly useful for confirming mutations in the TTR gene associated with ATTRv. WES allows the detection of rare mutations that could be overlooked with more traditional techniques or with the analysis of single genes. This technique can also be used to screen family members at risk of ATTRv, allowing early identification of carriers of the pathogenic variant.

Gene panels, which consist of the targeted analysis of a set of genes associated with certain diseases, are a rapid and relatively inexpensive technique for diagnosing cardiac amyloidosis when a specific genetic cause is suspected. The main advantage of gene panels lies in their accuracy and speed, with the ability to provide results in a short time, which is essential for immediately setting up an appropriate treatment.

WGS is the most comprehensive approach to analyzing all genetic variations in an individual, not only limited to coding regions such as WES but also including intronic regions, promoters, and other regulatory sequences that may be involved in the disease. Although WGS is more expensive and complex, it can be useful in cases of CA where WES or gene panels fail to identify pathogenic variants. This technique can uncover rare or unconventional mutations, such as structural rearrangements or copy number variations, that may play a role in the development of the disease.

The integration of WES, gene panels, and WGS with traditional clinical and instrumental methodologies, such as cardiac magnetic resonance imaging and scintigraphy, provides a comprehensive view of the patient’s clinical picture. The choice of the molecular technique to be used depends on the clinical suspicion, diagnostic urgency, and costs. However, in all cases, advanced genetic analysis allows for a more precise and early diagnosis, significantly improving therapeutic options, which can be personalized based on the patient’s genetic profile.

## 6. Therapeutic Strategies

### 6.1. Light Chain Amyloidosis

In AL amyloidosis, toxic light chains are responsible for cardiac damage, so treatment should aim mainly at their correction [73,74].

To choose the best therapeutic strategy, a risk classification of low, intermediate, or high is essential, which is based on several factors, such as the patient’s age; blood biomarkers such as NT-proBNP and troponin; and clinical symptoms such as the NYHA classification and Eastern Cooperative Oncology Group (ECOG) grade [75]. Additionally, the Mayo cardiac classification, which was used from 2004–2012, was developed to assess disease severity [76].

A promising strategy is autologous stem cell transplantation, which has excellent long-term results but is indicated only in selected cases [77]. Patients with low risk after Mayo staging revision, NYHA classification < III, age < 70 years, and biomarkers in the normal range are eligible for high-dose chemotherapy with melphalan after autologous stem cell transplantation [78].

The evolution of anti-plasma cell therapies has led to a more precise definition of the hematological response, and therapeutic options include alkylating agents, immunomodulators, corticosteroids, and proteasome inhibitors [79].

The combination of daratumumab with cyclophosphamide, bortezomib, and dexamethasone is indicated for patients with May 2004 stages I to IIIa disease [74]. In cases of insufficient response to therapy or existing organ damage, other combinations are used, such as melphalan and lenalidomide [75].

In Mayo stage IIIb patients, in a retrospective study examining 119 patients from different centers, the benefit of adding daratumumab to anti-plasma cell therapy was demonstrated [80]. Currently, daratumumab as a monotherapy for Mayo stage IIIb disease is currently being tested in a phase II trial [74].

### 6.2. Transthyretin Amyloidosis

Current therapies for ATTR amyloidosis include stabilizers, silencers, monoclonal antibodies, and therapies for the destruction of amyloid fibrils.

The main agent used is TTR silencing, which is involved in the synthesis of TTR in the liver, and the main drugs that silence TTR are as follows:

- Patisiran, which is a siRNA that reduces TTR levels by blocking the production of mRNA,
- Vutrisiran is an siRNA approved for polyneuropathy.
- Inotersen, which is an ASO that reduces TTR synthesis with improvements in left ventricular mass and exercise tolerance,
- Eplontersen is an ASO under study that has shown a significant increase in potency compared with Inotersen [74,81,82,83].

TTR stabilization prevents misfolding by binding to the tetramer, and the stabilizing drugs are as follows:

- Tafamidis, which binds the thyroxine binding site in the TTR tetramer, preventing dissociation into monomers and therefore amyloid formation, is approved for cardiac amyloidosis ATTR,
- Acoramidis mimics the protective T119M mutation by reducing TTR tetramer dissociation,
- Diflunisal of an NSAID that stabilizes the TTR tetramer by binding to the T4 binding site, which leads to improvements in cardiac biomarkers but is associated with side effects,
- Epigallocatechin-3-gallate (EGCG), a green tea extract that binds to the surface of the TTR tetramer, appeared to reduce myocardial mass in an observational study [74,84,85].

TTR destruction ensures the elimination of amyloid from the body by causing the amyloid fibril to break; this process involves the use of drugs such as doxycycline and TUDCA (doxycycline, tetracycline antibiotic, and tauroursodeoxycholic acid) [74,86].

Finally, immunotherapy, especially monoclonal antibodies (mAbs), could revolutionize the treatment of ATTR cardiomyopathy, not only by removing amyloid deposits in the heart but also by enhancing the immune response to aid in the recovery of cardiac function [87].

### 6.3. Heart Transplant

Cardiac transplantation is indicated in AL cardiac amyloidosis patients who respond to light chain suppressive therapies and in cases of terminal heart failure due to ATTR cardiac amyloidosis [73,88,89].

In the past, AL amyloidosis was a contraindication for transplantation due to high mortality [90]. Although new therapies have made this option possible, patients with cardiac amyloidosis have higher mortality on the waiting list than those with other cardiomyopathies do [91,92]. Furthermore, in patients with other variants, such as Thr60Ala, a double heart-liver transplant is indicated [73,79].

Table 4 summarizes the main therapies.

## 7. Current Challenges and Future Prospects

CA research has made much progress in recent years, yet there are still numerous significant challenges to diagnosis and therapy.

One of the main challenges is late diagnosis, as the symptoms of CA are often non-specific and are confused with other cardiac pathologies; it is therefore essential to improve awareness among doctors and promote differential diagnosis.

Another aspect is epidemiological underestimation; in particular, ATTRwt is probably much more frequent, but the diagnostic difficulty contributes to a lower diagnosis rate, which means that many cases may go undiagnosed or be discovered only in advanced stages, when therapeutic options are now limited.

The management of elderly patients is very complex; in fact, they often have comorbidities that require a multidisciplinary approach to balance specific therapies for amyloidosis with the treatment of other chronic pathologies to ensure an adequate quality of life.

Despite advances in therapies, CA is a progressive disease; current therapies slow the progression of the disease but are not yet able to reverse the cardiac damage that has already occurred.

Another challenge is represented by the high costs of new treatments, which are often prohibitive and not always accessible to all patients, especially in countries with limited healthcare resources, creating disparities in the treatment of patients worldwide.

The future prospects for CA require continuous research, early diagnosis, and equitable access to therapies, thus improving the quality and life expectancy of patients.

## 8. Conclusions

CA is a complex and potentially lethal disease, and a multidisciplinary approach involving cardiologists, hematologists, geneticists, neurologists, and pathologists is essential to personalize the therapeutic pathway, considering the different types of cardiac amyloidosis.

This tailored approach, which integrates advanced knowledge on molecular mechanisms and diagnostic accuracy, allows us to maximize the effectiveness of available therapies and significantly improve the quality of life and survival of patients.

## Figures and Tables

**Table 1:** Red flags in cardiac amyloidosis.

Light Chain Amyloidosis	Transthyretin Amyloidosis
Clinical red flags	Clinical red flags
Diastolic dysfunction (unspecific symptoms): asthenia, syncope, dizziness, orthostasis, and usually with preserved ejection fraction. Renal failure with proteinuria, hepatomegaly, autonomic neuropathy, sensorimotor peripheral neuropathy, gastrointestinal symptoms: more frequent extracardiac manifestations. Macroglossia and periorbital purpura: pathognomonic signs in advanced stages of the disease.	Diastolic dysfunction (unspecific symptoms): asthenia, syncope, dizziness, orthostasis, and usually with preserved ejection fraction. Carpal tunnel syndrome, especially if bilateral, rupture of the long head of the biceps, lumbar canal stenosis, sensorimotor peripheral neuropathy, vitreous opacity, cataract: extracardiac manifestations specific to ATTRwt.
Laboratory test red flags	Laboratory test red flags
Cardiac troponin: increase in serum values even before ventricular hypertrophy and higher than ATTR. Natriuretic peptides such as BNP and NT-proBNP: unexplained and disproportionate increase in serum levels with respect to the hemodynamic state.	Cardiac troponin: absent in the early stages and increase in serum levels subsequently in a manner directly proportional to infiltration. Natriuretic peptides such as BNP and NT-proBNP: unexplained and disproportionate increase in serum levels with respect to the hemodynamic state.
Electrocardiographic red flags	Electrocardiographic red flags
Low voltage EKG (more common in AL): reduction in QRS wave amplitude indicating amyloid infiltration of the heart. EKG with pseudoinfarct pattern: prominent Q waves due to amyloid deposition in the left ventricular wall. Conduction disturbances, especially atrioventricular blocks, and arrhythmias, especially atrial fibrillation.	Low voltage EKG: reduction in QRS wave amplitude indicating amyloid infiltration of the heart. EKG with pseudoinfarct pattern: prominent Q waves due to amyloid deposition in the left ventricular wall. Conduction disturbances, especially atrioventricular blocks, and arrhythmias, especially atrial fibrillation.
Red flags in cardiac imaging	Red flags in cardiac imaging
Echocardiography: ventricular thickening with reduction in cavity dimensions, predominantly concentric pattern, up to restrictive cardiomyopathy.	Echocardiography: ventricular thickening with reduction in cavity dimensions, septal asymmetric pattern, up to restrictive cardiomyopathy. Positive diphosphonate scintigraphy: detects amyloid deposition without the need for biopsies.

**Table 2:** Diagnostic steps for cardiac amyloidosis.

Step	Procedure	Notes
*1. Clinical evaluation*	Clinical symptoms: cardiac failure, arrhythmias, etc.	Consider the presence of clinical signs suggesting cardiac amyloidosis.
*2. Imaging and functional tests*	Echocardiography: evaluate typical echocardiographic signs. Bone scintigraphy with diphosphonate: evaluate the absorption of the radiotracer.	Gradual absorption 2 or 3: suggests cardiac amyloidosis ATTR.
*3. Histopathological*	Periumbilical subcutaneous tissue biopsy, and if necessary, endomyocardial biopsy. Congo Red to highlight amyloid deposits: - Red-orange color: observable under an optical microscope; - Apple-green birefringence: observable under a polarized light microscope. For amyloid subtyping: - Immunohistochemistry: antibodies against TTR, AA, lambda, and kappa to visualize amyloid deposits in tissue sections, - Immunofluorescence: antibodies labeled with fluorescent dyes to detect amyloid deposits in frozen sections, - Immunologically Labeled Electron Microscopy: combines IHC and electron microscopy, using gold-labeled antibodies to identify proteins in fibrils, - Laser microdissection and tandem mass spectrometry: analyzes amyloid deposits from Congo Red-stained paraffin sections using mass spectrometry.	Subcutaneous tissue biopsy: minimally invasive, with lower sensitivity. Endomyocardial biopsy: invasive, with higher sensitivity. Limitations of histochemical staining: beware of false positives from nonamyloid tissue components. Limitations: - IHC limited by availability of antibodies and variable sensitivity and specificity, - IF limited by variable results, - IEM limited by the availability of gold-labeled antibodies and the need for highly specialized skills, - LCM-MS limited by availability of advanced equipment and specialized skills.
*4. Nonbiopsy diagnostic criteria for ATTR*	If there are typical echocardiographic signs and gradual 2 or 3 absorption on bone scan.	The diagnosis of ATTR cardiac amyloidosis can be made without biopsy.
*5. Exclusion of other conditions*	Free light chain assay: check for abnormal levels. Electrophoresis of serum and urinary proteins with immunofixation: exclude monoclonal proteins.	Exclude clonal dyscrasias or other conditions that may mimic amyloidosis.
*6. Diagnosis*	Integrate biopsy results with clinical, imaging, and biochemical data for accurate diagnosis.	If biopsy has been performed: confirm with Congo Red staining. If using imaging and nonbiopsy criteria: Confirm ATTR cardiac amyloidosis based on clinical and imaging findings.

**Table 3:** Molecular mechanisms of cardiac amyloidosis.

	Light Chain Amyloidosis	Transthyretin Amyloidosis
*Causes*	Monoclonal immunoglobulin light chains produced by plasma cells, mostly λ chains.	Pathogenic variants in ATTRv or age-related instability in ATTRwt.
*Mechanisms*	Internalization into cardiomyocytes and fibroblasts, oxidative stress, mitochondrial ultrastructural changes, activation of apoptosis (p38 MAPK pathway), increased MMP9/TIMP-1, impairment of ventricular relaxation and contractile function.	TTR tetramer instability, dissociation into monomers, oxidative stress, cardiomyocyte apoptosis, increased basement membrane components, and amyloid deposition.
*Alterations*	Heart: impairment of ventricular relaxation, myocardial fibrosis, and apoptosis.	Heart: restrictive cardiomyopathy and neuropathic or mixed signs.

**Table 4:** Treatment.

	Light Chain Amyloidosis	Transthyretin Amyloidosis
*Indications*	The main goal is the correction of toxic light chains.	Stabilization and silencing of TTR to prevent the formation of amyloid fibrils.
*Therapy*	Autologous stem cell transplant (in selected patients) High-dose chemotherapy with melphalan Anti-plasma cell therapies: • Alkylating agents; • Immunomodulators; • Corticosteroids. Proteasome inhibitors Heart transplant in AL cardiac amyloidosis with response to light chain suppressive therapies	Silencing: • Patisiran (siRNA); • Vutrisiran (siRNA); • Inotersen (ASO); • Eplontersen (ASO). TTR stabilization: • Tafamidis; • Acoramidis; • Diflunisal; • EGCG. TTR destruction: • Doxycycline + TUDCA (tetracycline antibiotic + tauroursodeoxycholic acid) Heart transplant if end-stage heart failure
*Notes*	Risk classification into low, intermediate, and high, based on biomarkers NT-proBNP and troponin, age, clinical symptoms NYHA and ECOG. Mayo Stages I-IIIa: Combined treatment with daratumumab, cyclophosphamide, bortezomib, and dexamethasone. Mayo Stage IIIb: ongoing study of daratumumab as monotherapy.	Immunotherapy (mAbs) under study to remove amyloid deposits and enhance the immune response. Combined heart–liver transplantation in patients with genetic variants.
